# Safety and efficacy of desmopressin (DDAVP) in preventing hematoma expansion in intracranial hemorrhage associated with antiplatelet drugs use: A systematic review and metaanalysis

**DOI:** 10.1002/brb3.3540

**Published:** 2024-05-23

**Authors:** Faizan Shahzad, Usman Ahmed, Ayesha Muhammad, Farhan Shahzad, Syed Imam Naufil, Mohamad Walid Sukkari, Abdullah Bin Kamran, Sara Murtaza, Marwah Bintay Khalid, Haroon Shabbir, Sajeel Saeed

**Affiliations:** ^1^ Medical Student Rawalpindi Medical University Rawalpindi Pakistan; ^2^ Department of Medicine Holy Family Hospital Rawalpindi Pakistan; ^3^ Faculty of Medicine University of Aleppo Aleppo Syrian Arab Republic

**Keywords:** desmopressin, hematoma expansion, intracerebral hemorrhage, neurocritical care

## Abstract

**Introduction:**

One of the most serious complications associated with antiplatelet agents is antiplatelet‐associated intracranial hemorrhage (AA‐ICH). Desmopressin is a synthetic antidiuretic hormone (ADH) analog. It has been linked to improving patient outcomes in antiplatelet‐induced intracranial hemorrhage. The secondary outcomes included the incidence of thrombotic complications and neurological outcomes.

**Methods:**

A systematic search was conducted on three databases (PubMed, Cochrane, and ClinicalTrials.gov) to find eligible literature that compares desmopressin (DDAVP) versus controls in patients with AA‐ICH. The Mantel–Haenszel statistic was used to determine an overall effect estimate for each outcome by calculating the risk ratios and 95% confidence intervals (CI). Heterogeneity was measured using the *I* ^2^ test. The risk of bias in studies was calculated using the New Castle Ottowa Scale.

**Results:**

Five studies were included in the analysis with a total of 598 patients. DDAVP was associated with a nonsignificant decrease in the risk of hematoma expansion (RR = .8, 95% CI,.51–1.24; *p* = .31, *I* ^2^ = 44%). It was also associated with a non‐significant decrease in the risk of thrombotic events (RR,.83; 95% CI,.25–2.76; *p* = .76, *I* ^2^ = 30%). However, patients in the DDAVP group demonstrated a significant increase in the risk of poor neurological outcomes (RR, 1.31; 95% CI, 1.07–1.61; *p* = .01, *I* ^2^ = 0%). The risk of bias assessment showed a moderate to low level of risk.

**Conclusion:**

DDAVP was associated with a nonsignificant decrease in hematoma expansion and thrombotic events. However, it was also associated with a significantly poor neurological outcome in the patients. Thus, until more robust clinical trials are conducted, the use of DDAVP should be considered on a case‐to‐case basis.

## Graphical abstract

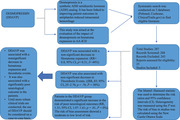

## 1 INTRODUCTION

Desmopressin; an ADH analog and hemostatic agent, in recent years, has become incredibly relevant in the intracranial hemorrhage (ICH) landscape (Keep et al., 2012), specifically when discussing ICH due to antiplatelet therapy.

**FIGURE 1:**
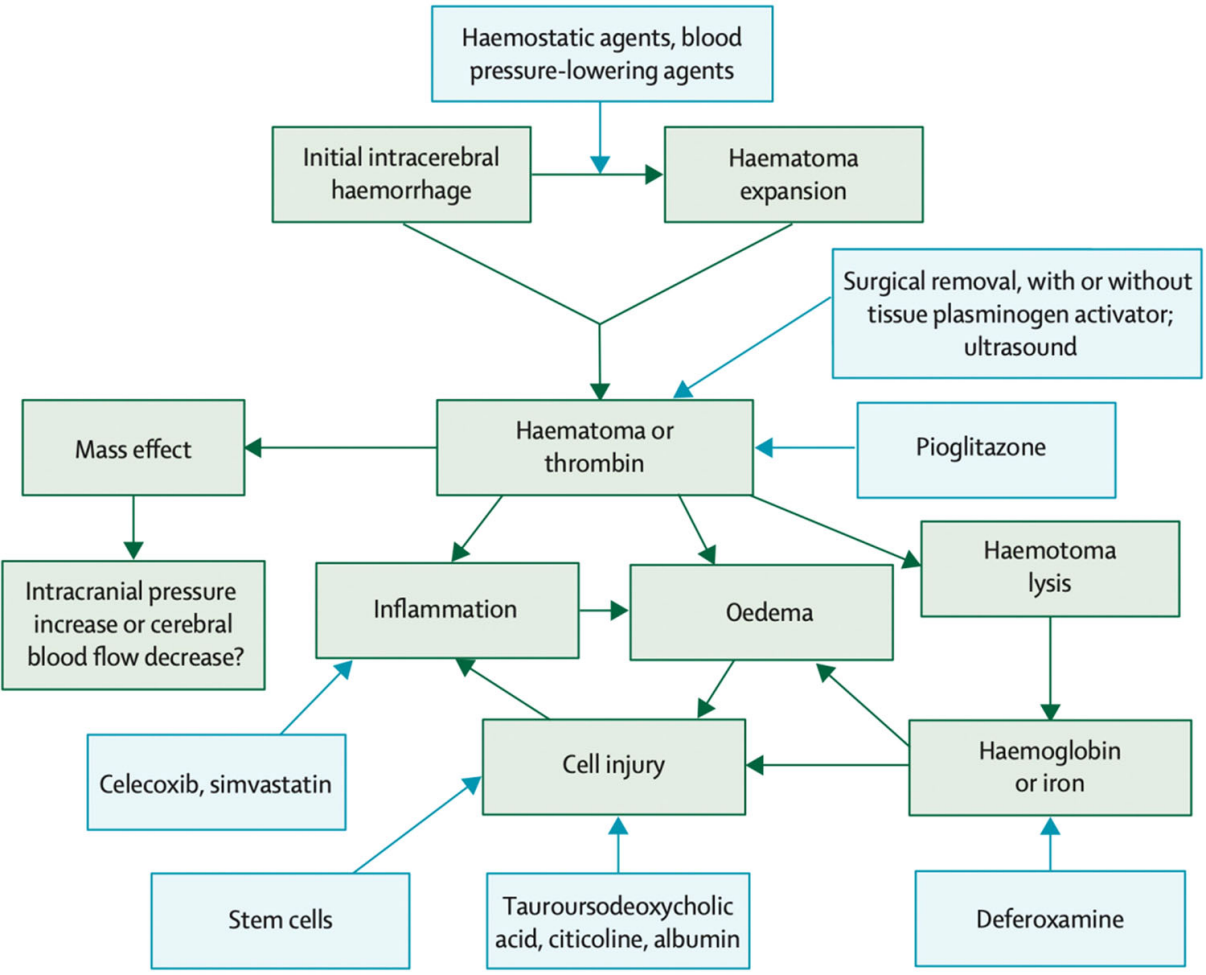
The possible pathologies of intracranial hemorrhage (ICH) and therapeutic targets (Keep et al., 2012).

Different pathologies of ICH and their respective therapeutic targets have been described (Keep et al., 2012) (Figure 1).

It is common knowledge that thrombotic events, such as myocardial infarction, ischemic stroke, and peripheral artery disease, significantly affect global morbidity and mortality rates (Capodanno et al., 2019). These conditions arise from the formation of arterial thrombi (Asada et al., 2018), predominantly composed of platelets, which obstruct blood flow and compromise tissue perfusion. Antiplatelet drugs play a crucial role in managing this risk by modulating platelet function to reduce the severe consequences of thrombotic events (Thachil, 2016).

The rationale behind antiplatelet agents lies in their capacity to hinder platelet activation and aggregation, thus impeding the formation of thrombi and diminishing the likelihood of thrombotic complications. However, despite the benefits of antiplatelet therapy in cardiovascular events like acute coronary syndrome (Layne & Ferro, 2017), there are drawbacks to consider.

One of the most serious complications associated with these agents is antiplatelet‐associated ICH (AA‐ICH) (Caceres & Goldstein, 2012; Ha et al., 2021), a condition characterized by bleeding within the brain parenchyma or surrounding structures (Caceres & Goldstein, 2012). The primary mechanism leading to AA‐ICH involves the disruption of the delicate balance between hemostasis and bleeding (Alamin et al., 2022). Antiplatelet drugs inhibit platelet activation and aggregation through various pathways, such as cyclooxygenase‐1 inhibition (aspirin) and adenosine diphosphate receptor antagonism (clopidogrel) (Gachet, 2015; Hilkens et al., 2021). In addition, the long half‐life of these drugs prevents their timely clearance in the case of a need for reversal of their effects (Bultas, 2013). Although this effect is desirable in preventing arterial thrombosis, it also impairs the formation of stable clots in response to vessel wall injury.

Consequently, AA‐ICH can occur due to weakened platelet plug formation and compromised clot stabilization, resulting in increased vulnerability to spontaneous bleeding in the brain's vasculature (Alter et al., 2020). AA‐ICH poses significant challenges in clinical practice. Patients with preexisting risk factors, such as advanced age, hypertension, and coagulopathy, are particularly susceptible (Zeng et al., 2022). The severity of **AP‐ICH can vary, ranging from minor microbleeds to life‐threatening hemorrhages causing neurological deficits or death.

Existing literature has established that antiplatelet therapy increases the risk of hematoma expansion in ICH (Law et al., 2021). In the setting of intracranial hemorrhage, antiplatelet therapy has been reported to be associated with larger baseline hematoma volume when compared to patients that were not previously on antiplatelet therapy (Camps‐Renom et al., 2017), increased risk of intraventricular hemorrhage (Naidech et al., 2012), and hematoma expansion (Burchell et al., 2017). However, other studies have contradicted these findings, showing that antiplatelet therapy does not have significant effects on hematoma characteristics (Sansing et al., 2009). Other potential treatment modalities for AA‐ICH such as platelet transfusions and tranexamic acid have variable outcomes, where platelet transfusions increased the odds of death or dependence (Baharoglu et al., 2016), but the use of tranexamic acid significantly reduced hematoma expansion (Law et al., 2021). Desmopressin influences the body's vasopressin receptors and finds application in various clinical contexts, encompassing conditions like diabetes insipidus, enuresis, and hemophilia (Enna & Bylund, 2011).

In addition, desmopressin has been linked to ameliorating patient outcomes in two critical scenarios. First, in treating antiplatelet‐induced intracranial hemorrhage (Mohinani et al., 2023). Second, trauma resuscitation involving active hemorrhage, where can aid in managing the balance between coagulation and bleeding control (Mohinani et al., 2023). This utility stems from the role of desmopressin in causing exocytosis of von Willebrand Factor and Factor VIII from storage sites, taking the first step toward thrombogenesis (Mohinani et al., 2023).

Although the potential of desmopressin remains an area of interest, the existing body of literature highlights the necessity for a measured and evidence‐based approach to its incorporation in the intricate domain of AA‐ICH. The primary objective of this systematic review and meta‐analysis is to consolidate and evaluate the impact of desmopressin on the occurrence and outcomes of antiplatelet‐associated intracerebral hemorrhage. This comprehensive approach seeks to provide a clearer understanding of how desmopressin affects this specific type of intracranial hemorrhage, offering insights that can guide clinical decision‐making and patient care.

## 2 METHODS

### 2.1 Search strategy

This meta‐analysis followed the PRISMA guidelines (Page et al., 2021) and a systematic search of the literature was conducted on July 27, 2023. The authors searched databases, including PubMed, Cochrane, and ClinicalTrials.gov without any time restrictions. The search included all MesH terms related to antiplatelet, intracranial hemorrhage, and desmopressin (DDAVP). To find articles that were not found during the first screening, the reference lists of relevant articles were examined using backward snowballing. The literature search did not have any time restrictions.

### 2.2 Eligibility criteria

The authors included randomized controlled trials, cohort studies, case–control studies, and case series that assessed the effect of desmopressin (DDAVP) on patients with intracranial hemorrhage undergoing antiplatelet therapy. Conference abstracts with sufficient data on the outcomes and variables were also included. The authors excluded case reports, review articles, editorials, and non‐English studies.

### 2.3 Study selection

Authors UA and FS independently reviewed titles, abstracts, and full texts of all the articles that were sought after the systematic search. The inclusion criteria for the study were as follows: (1) The population consisted of adults with spontaneous ICH who had documented use of antiplatelet therapy before admission, (2) interventions that compared DDAVP to controls, and (3) at least one or more clinical outcomes focused on hematoma expansion, thrombotic events, neurological outcomes, and decrease in serum sodium. Thrombotic events included deep vein thrombosis (DVT), pulmonary embolism, myocardial infarction, and ischemic stroke. Both randomized clinical trials and controlled observational cohort studies were included in the review. They selected five studies that met the inclusion criteria and excluded the irrelevant studies. Any disagreements between these two authors were resolved through group discussions with a third investigator (A.M.).

### 2.4 Data extraction and quality assessment

A data collection sheet of all the important outcomes and relevant variables was made on Google Sheets. The reviewers recorded characteristics of the studies and populations, including first author, publication year, sample size, age, gender, initial ICH volume, initial systolic blood pressure (SBP), and baseline serum sodium level. The authors used Review Manager v5.4.1 for the analysis and assessed the quality of observational studies using the Newcastle‐Ottawa scale (Stang, 2010; Wells et al., 2000).

### 2.5 Outcomes and statistical analysis

The primary outcome was hematoma expansion defined as an increase in hematoma volume greater than or equal to 20% within 24 h after administration of DDAVP. The secondary outcomes were thrombotic events up to 90 days or at discharge, and poor neurological outcome modified Rankin Scale (mRS) greater than or equal to 4 at 90 days or discharge. Outcomes of efficacy included hematoma expansion and neurological outcome, whereas the outcome of safety included thrombotic events.

The Mantel–Haenszel method was used to calculate an overall, effect estimate of treatment with DDAVP for each specific outcome by combining the stratum‐specific risk ratios (RRs). A *z*‐test was used to determine the significance of the RR. The *I* ^2^ was calculated using a *v*2 test to assess variability due to heterogeneity rather than chance, with substantial heterogeneity assumed when *I* ^2^ > 50%. The 95% confidence interval (CI) for hematoma expansion, thrombotic events, and poor neurological outcomes was calculated using the Wilson method and displayed in forest plots. A *p*‐value <.05 was considered statistically significant. Statistical analysis was performed using Review Manager 5.4.1.

## 3 RESULTS

### 3.1 Search results

**FIGURE 2:**
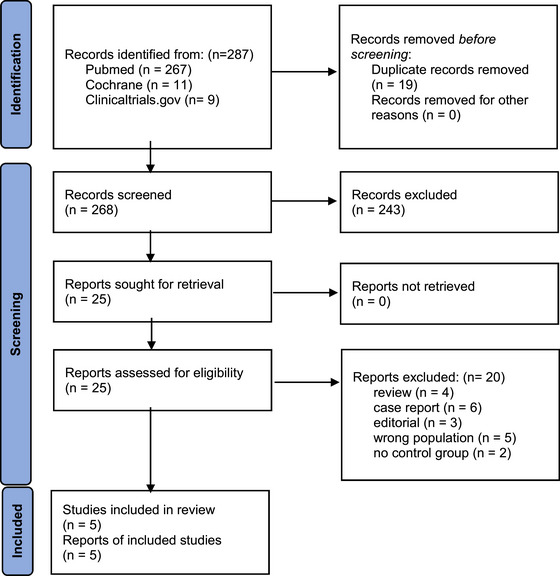
PRISMA 2020 flow diagram for literature review. This figure summarizes the process of article screening (including both primary and secondary screening). There were a total of 287 articles from the systematic search. Of these, only five met the inclusion criteria and were included in the study.

A systematic search was conducted on July 27, 2023, which yielded a total of 287 articles. After the removal of 19 duplicate articles, the titles and abstracts of 268 articles were read. During the primary screening, 243 articles were excluded. The full texts of the remaining 25 articles were retrieved and read. A total of 20 studies were excluded during the secondary screening. Data from five studies was collected which included four nonrandomized retrospective studies (including one retrospective chart review, one retrospective cohort, and two retrospective studies), and one randomized phase two clinical trial. The literature selection process is summarized in Figure 2.

### 3.2 Study characteristics

A total of 5 studies were included in the analysis, comprising 598 patients. The patients were divided into two groups: DDAVP and Control. The DDAVP group had a total of 301 patients and the control group had a total of 297 patients. The studies were published between 2019 and 2023. The sample size ranged from 54 to 209. The mean age in the DDAVP group was 71–76.4, and the mean age in the control group was 71–76. The DDAVP group had a total of 179 males (59.4%), and the control group had a total of 180 males (60.6%). The median initial ICH volume in the DDAVP group ranged from 3.9 to 45.4 mL and that in the control group ranged from 6.1 to 36.1. The median initial SBP ranged from 145.5 to 169 in the DDAVP group and 148.1 to 163 in the control group. Lastly, median baseline serum sodium values ranged from 135.6 to 139 in the group that was administered DDAVP and from 138 to 141 in the control group. We have summarized the baseline characteristics in Table 1.

**TABLE 1:** Table of the baseline characteristics of the studies included in the analysis.

Study characteristics	Study, author, and year					
	Study groups	Desborough 2023	Feldman 2019	Mengel 2020	Schmidt 2019	Summers 2023
Study design	–	Phase 2 trial	Retrospective chart review	Retrospective study	Retrospective cohort	Retrospective study
Sample size (*n*)	DDAVP	27	55	72	29	118
	Control	27	69	68	42	91
Age (years), mean ± (SD)	DDAVP	76.4 (10.7)	72 (67.0–81.0)	73 (15.5)	71 (12.5)	73 (Camps‐Renom et al., 2017)
	Control	72.7 (11.7)	73.5 (63.5–82.0)	72 (16.3)	76 (11.4)	71 (Alter et al., 2020)
Gender males *n* (%)	DDAVP	16 (59%)	29 (52.7%)	44 (61.1%)	17 (59%)	73 (61.9%)
	Control	20 (74%)	34 (49.2%)	45 (66.1%)	24 (57%)	57 (62.6%)
Initial ICH volume (mL) median (IQR)	DDAVP	13.3 (4.4–32.0)	17.0 (4.5–54.6)	45.4 (47)	32.1 (58.3)	3.9 (1.1–11.6)
	Control	8.1 (3.3–17.0)	25.0 (4.2–76.1)	36.1 (55.1)	25.7 (43.2)	6.1 (1.8–22.6)
Initial SBP (mmHg) median ± (SD)	DDAVP	151.5 (19.8)	145.5 (27.5)	169 (62)	165 (Gierisch et al., 2014)	147 (128–169)
	Control	151.9 (24.4)	148.1 (27.9)	163 (62)	157 (Stang, 2010)	151 (132–176)
Baseline serum sodium (mEq/L)	DDAVP	135.6 (3.6)	139.0 (136–141)	N/R	N/R	138 (135–139)
	Control	138.1 (4.2)	141 (138.5–143)	N/R	N/R	138 (135–140)

*Note*: This table represents the baseline characteristics of participants in each of the included studies. The characteristics of the DDAVP and control group have been presented separately.

Abbreviations: DDAVP, desmopressin; ICH, intracranial hemorrhage; SBP, systolic blood pressure.

The new Castle Ottawa scale was used to assess the risk of bias in the selected studies which is the recommended bias assessment tool for controlled observational studies (Stang, 2010; Wells et al., 2000). According to the scale, 4 points are attributed to the selection of patients, 2 for comparability, and 3 for outcome. The total number of points that one study can have is 9 (Gierisch et al., 2014). The studies by Desborough et al., Mengel et al., and Summers et al. showed a low risk of bias with 9, 8, and 8 points, respectively, whereas Feldman et al. and Schmidt et al. showed a moderate risk of bias with 7 and 6 points, respectively. It was not possible to determine if there was publication bias through a visual examination of the funnel plot's asymmetry because there were fewer than 10 studies for each outcome. The risk of bias assessment table has been shown in Table 2.

**TABLE 2:** Risk of bias assessment using the New Castle Ottowa Scale.

Author name	Type of study	Year	Selection	Comparability	Exposure/outcome	Overall
**Desborough** (2023)	Randomized phase 2 trial	2023	****	**	***	9
Feldman et al. (2019)	Retrospective chart review	2019	***	*	***	7
Mengel et al. (2020)	Retrospective study	2020	***	**	***	8
**Schmidt** (2019)	Retrospective cohort	2019	**	*	***	6
Summers et al. (2023)	Retrospective study	2023	***	**	**	8

*Note*: The New Castle Ottowa Scale has a score total of 9, with 4 points for participant selection, 2 for comparability, and 3 for outcomes. A low risk of bias is at 8 or 9 points, a moderate risk of bias is at 6 or 7 points, and 5 or below 5 points are considered to be a high risk of bias. The studies by Desborough et al., Mengel et al., and Summers et al. showed a low risk of bias with 9.8, and 8 points, respectively, whereas Feldman et al. and Schmidt et al. showed a moderate risk of bias with 7 and 6 points, respectively.

### 3.3 Hematoma expansion

The outcome of efficacy was hematoma expansion, defined as an absolute increase in hematoma volume greater than or equal to 20% within 24 h after the administration of DDAVP (Law et al., 2021). All five studies measured the rate of hematoma expansion. Out of 298 total patients who received DDAVP, 55 showed hematoma expansion (18.4%); out of 292 patients in the control group, 73 showed hematoma expansion (25%). This showed a decrease in the risk of hematoma expansion in the DDAVP group; however, the result was insignificant (*p* = .31). A moderate amount of heterogeneity was noticed *I* ^2^ = 44% but it was insignificant (*p* = .13).

### 3.4 Subgroup analysis of hematoma expansion

To decrease the heterogeneity, the outcome was divided into two subgroups based on the percentage of hematoma expansion. The first subgroup used an increase of 20% from the baseline value as the definition of hematoma expansion. Feldman et al. and Summers et al. were included in this subgroup. Out of 173 patients who were administered DDAVP, 25 showed hematoma expansion (14.4%), and out of 160 patients in the control group, 41 showed hematoma expansion (25.6%). The risk ratio was 0.54 (RR = .54; 95% CI,.18–1.63; *p* = .28).

**FIGURE 3:**
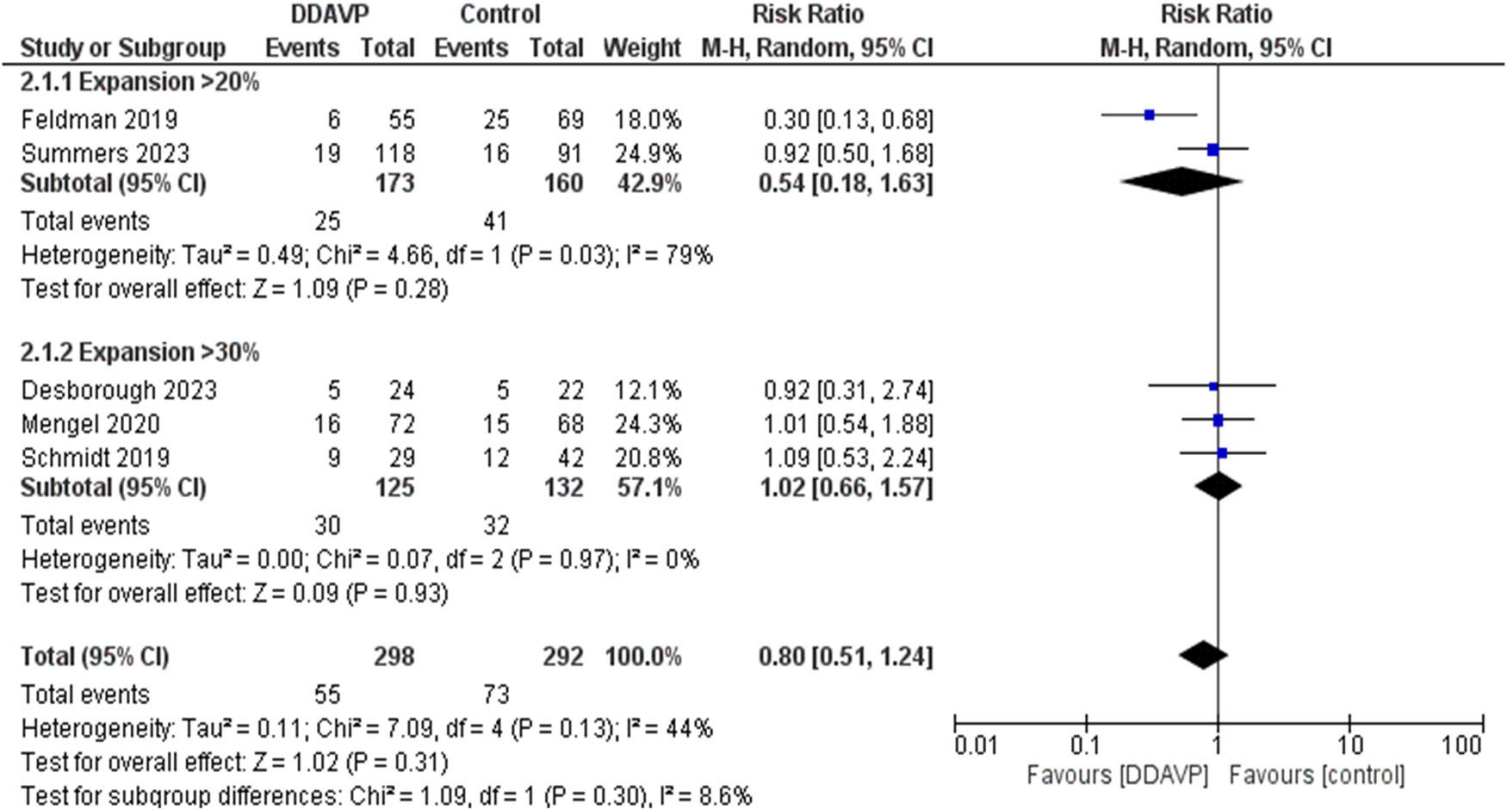
Forest plot comparing the effect of desmopressin (DDAVP) versus control on hematoma expansion. This forest plot pools the data for hematoma expansion. The subgroups have been made on the degree of hematoma expansion, that is, either more than 20% or more than 30%. Heterogeneity was assessed using an *I* ^2^ test. A *z*‐test was applied to test for the overall effect with a 95% confidence interval. Furthermore, the pooled analyses show a RR of.80 with *p*‐value = .31 and *I* ^2^ = 44%.

The second subgroup defined hematoma expansion as an increase of 30% from the baseline value within 24 h. It comprised data from three studies; Desborough et al., Mengel et al., Schmidt et al. Out of 125 patients in the DDAVP group; hematoma expansion occurred in 30 patients (24%) and out of 132 patients in the Control group, hematoma expansion occurred in 32 patients (24.2%). The risk ratio was 1.02 (RR, 1.02; 95% CI,.66–1.57; *p* = .93). Figure 3 summarizes these results.

### 3.5 Thrombotic events

**FIGURE 4:**
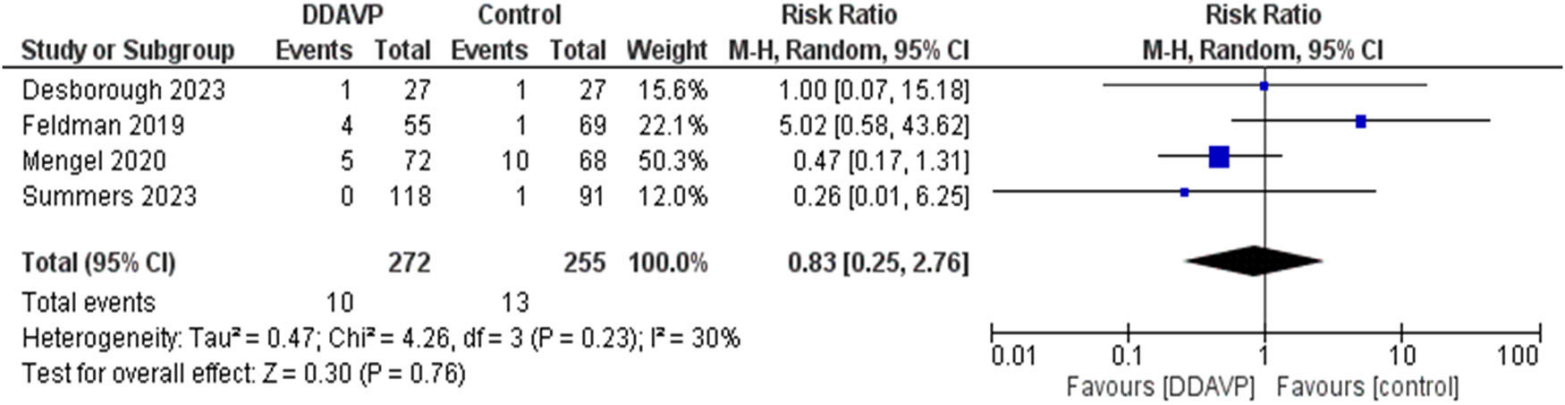
Forest plot showing the effect of desmopressin (DDAVP) versus control on thrombotic events. This forest plot pools the data for thrombotic events. Heterogeneity was measured using an *I* ^2^ test. A *z*‐test was applied to test of overall effect with a 95% confidence interval. It resulted in a RR of.83, *p*‐value = .76, and *I* ^2^ = 30%.

The outcome of safety was new onset thrombotic events up to 90 days. Four studies assessed the occurrence of thrombotic events. stroke, ischemic heart disease, myocardial infarction (NSTEMI), DVT, and pulmonary embolism were included as thrombotic events. Out of a total of 272 patients who received DDAVP, only 10 (3.6%) had thrombotic events and out of 255 patients in the control group, only 13 (5.1%) had thrombotic events. The Risk ratio was.83 (RR,.83; 95% CI,.25–2.76; *p* = .76). These results showed that DDAVP was correlated with a lower occurrence of thrombotic events, however, the results were insignificant (*p* = .76). The results showed a moderate heterogeneity of *I* ^2^ = 30%. These results have been summarized in Figure 4.

### 3.6 Neurological outcome

Poor neurological outcome was defined as a score of 4–6 on the modified Rankin Scale. Three studies evaluated the neurological outcomes. Out of a total of 128 patients who were administered DDAVP, 81 (63.3%) showed poor neurological outcomes, and out of 137 patients in the control group, 68 (49.6%) showed poor neurological outcomes. The risk ratio was 1.31 (RR, 1.31; 95% CI, 1.07–1.61; *p* = .01). These results show a significant increase in the risk of poor neurological outcomes in the DDAVP group. No heterogeneity was detected in the results *I* ^2^ = 0%.

### 3.7 Subgroup analysis of poor neurological outcome

Neurological outcome was measured either at 90 days or at discharge for which two separate subgroups were created. The first subgroup included data from Desborough et al. and Mengel et al. and measured neurological outcomes at discharge. Out of 99 patients in the DDAVP group, 58 (58.6%) showed poor neurological outcomes and out of 95 patients in the control group, 43 (45.2%) showed poor neurological outcomes. The risk ratio was 1.29 (RR, 1.29; 95% CI,.98–1.71; *p* = .07).

**FIGURE 5:**
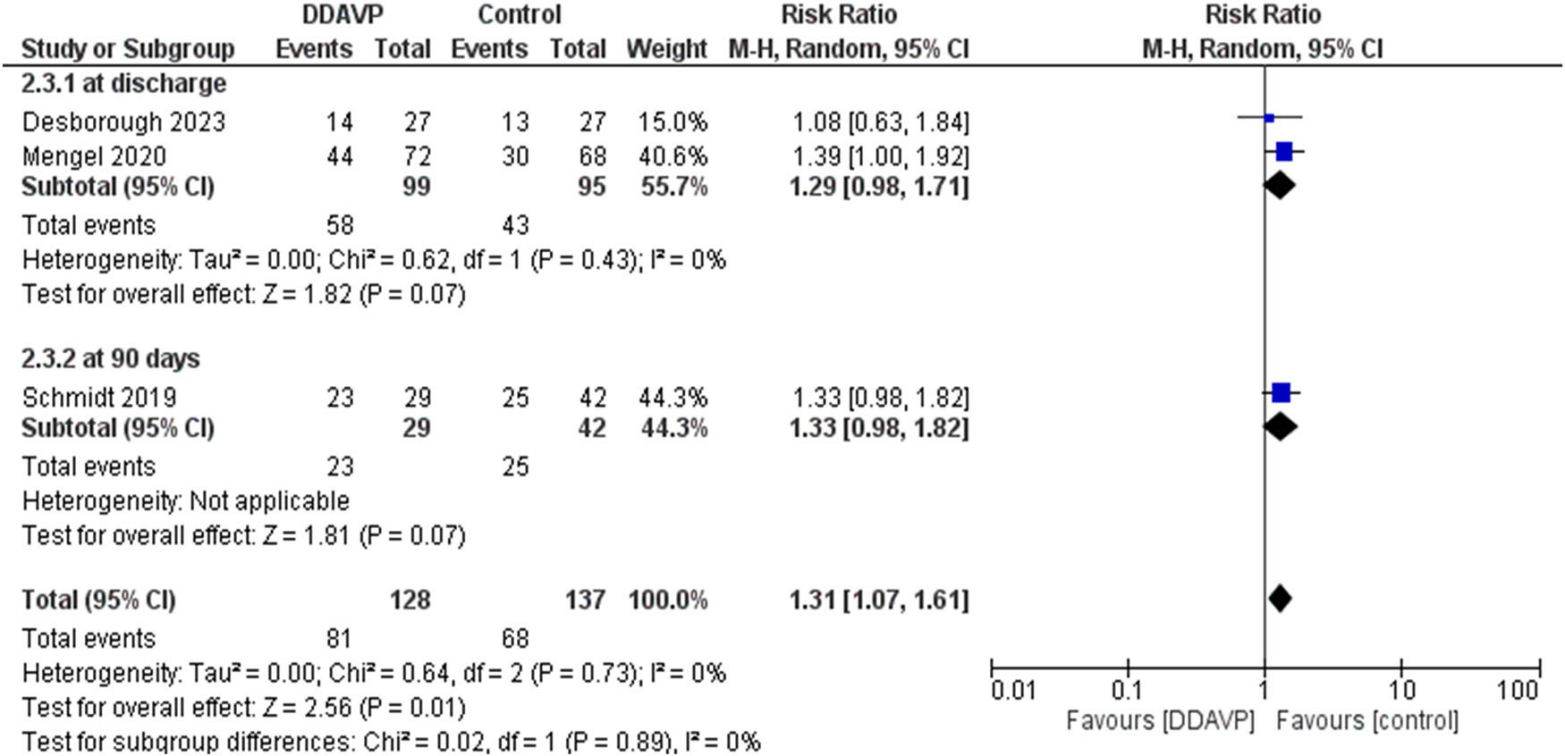
Forest plot comparing the effect of desmopressin (DDAVP) versus control on poor neurological outcome (mRS ≥ 4) This forest plot pools the data for neurological outcome. A modified Rankin Scale (mRS) was used. A worse neurological outcome was identified at an mRS score of 4 or higher. The data was subdivided into two groups, that is, either mRS was calculated at discharge or 90 days from initial hospital admission. Heterogeneity was measured using an *I* ^2^ test. A *z*‐test was applied to test of overall effect with a 95% confidence interval. It resulted in a RR of 1.31, *p*‐value = .01, *I* ^2^ = 0%.

The second subgroup consists of data from Schmidt et al. and measured neurological outcomes at 90 days. Out of 29 patients in the DDAVP group, 23 (79.3%) showed events and out of 42 patients in the control group, 25 (59.5%) showed events. The risk ratio was 1.33 (RR, 1.33; 95% CI,.98–1.82; *p* = .07). These results have been summarized in Figure 5.

## 4 DISCUSSION

The findings of this meta‐analysis contribute insights into the complex relationship between DDAVP and antiplatelet‐associated ICH. Although the study is unable to conclusively establish DDAVP as the preferred medication for controlling AA‐ICH, the analysis of hematoma expansion, a primary outcome, showed a trend toward lower risk in the DDAVP group compared to the control group. Out of 298 total patients who received DDAVP, 18.4% showed hematoma expansion, whereas 25% of patients in the control group showed hematoma expansion. This shows a decrease in the risk of hematoma expansion in the DDAVP group; however, the result was insignificant. Although this reduction was not statistically significant, it does warrant investigation, as it may contribute to the Neurocritical Care Guidelines (Frontera et al., 2016).

In terms of safety, DDAVP appeared to be associated with a lower occurrence of thrombotic events. Out of a total of 272 patients who received DDAVP, only 3.6% had thrombotic events and out of 255 patients in the control group, only 5.1% had thrombotic events. Although the observed effect did not reach statistical significance, it raises the possibility that DDAVP might influence the thrombotic risk profile in patients with antiplatelet‐associated ICH.

On the other hand, the increased risk of poor neurological outcomes associated with DDAVP administration is a concerning finding, suggesting that cautious consideration is necessary when evaluating the therapeutic potential of DDAVP in this context. Although the statistical significance of certain outcomes remains elusive, the trends observed highlight the potential of DDAVP as a therapeutic intervention for antiplatelet‐associated ICH. These insights guide clinicians in assessing the feasibility and safety of integrating DDAVP into treatment strategies. The heightened risk of poor neurological outcomes prompts careful consideration and underscores the need for personalized approaches that consider individual patient profiles and risk factors.

The secondary outcomes included intra‐ventricular hemorrhage, hydrocephalus, thromboembolic events, and functional outcomes assessed by the modified Rankin Scale at 3 months. No significant differences were observed between the treatment group and controls in these aspects. Even among patients with substantial hematoma expansion, the administration of 1‐deamino‐8‐d‐arginine vasopressin and platelet transfusion did not yield a considerable advantage.

Comparing these findings with previous research in the field highlights both consistencies and discrepancies. Although some studies like Feldman et al. (2019) have reported potential benefits of DDAVP in reducing hematoma expansion and thrombotic events, the meta‐analysis results do not uniformly support these claims.

Summers et al. (2023) noted that although recommended by SCCM guidelines (Society of Critical Care Medicine Guidelines (SCCMC)), DDAVP warrants scrutiny to establish its efficacy in mitigating this expansion risk. The study's findings indicate similar expansion rates between DDAVP and non‐DDAVP groups. Secondary outcomes involve thrombotic complications within 7 days of the event. With DDAVP's pro‐hemostatic properties (Mohinani et al., 2023), concerns arise about tipping the balance toward thrombosis, compounding antiplatelet‐associated risks. However, this study's result of comparable thrombotic complication rates in both groups aligns with the idea that DDAVP's hemostatic effects might not significantly increase thrombosis risk. Beyond immediate clinical implications, the study encourages longer term functional outcome investigations. It implies that the minimal benefit or harm of DDAVP necessitates more comprehensive exploration.

Similarly, Mengel et al. (2020) stated that despite the theoretical potential of DDAVP, the study results indicate no significant reduction in hematoma expansion when compared to controls. The primary outcome, relative hematoma expansion from baseline to follow‐up CT, showed no substantial between‐group differences. This result aligns with the findings of a previous randomized trial (Platelet Transfusion Versus Standard Care After Acute Stroke Due to Spontaneous Cerebral Hemorrhage Associated with Antiplatelet Therapy trial) (Baharoglu et al., 2016) reinforcing the limited hemostatic efficacy of early platelet transfusion in antiplatelet‐associated ICH.

However, Feldman et al. (2019) found that DDAVP administration was associated with a significant decrease (88%) in the likelihood of intracranial hemorrhage expansion during the initial 24 h compared to the non‐DDAVP group. Despite this positive impact on hemorrhage expansion, DDAVP did not significantly affect the largest median absolute decrease in serum sodium, even though it has been observed to concentrate urinary sodium to prevent rapid sodium correction in past studies (Rafat et al., 2014). Neither did it affect the occurrence of thrombotic events during the study period. The mixed outcomes emphasize the complexity of the underlying mechanisms and the variability inherent in in‐patient populations.

Acknowledging the limitations of this meta‐analysis is crucial for contextualizing the findings. The predominance of retrospective studies and the relatively small sample sizes introduce inherent biases and hinder the ability to draw definitive conclusions. Heterogeneity among the included studies further complicates the interpretation of results. Although the findings offer intriguing insights into hematoma expansion, thrombotic events, and neurological outcomes, the lack of statistical significance and the increased risk of poor neurological outcomes emphasize the need for further investigation. Moreover, a lack of clinical evidence and the availability of only preliminary experimental data in this study hinders the generalization of the results. Future research endeavors should prioritize well‐designed, prospective randomized controlled trials to provide more robust evidence. Exploring optimal dosages, patient‐specific factors, and potential interactions with different antiplatelet agents will be pivotal in shaping future treatment paradigms.

## 5 CONCLUSION

DDAVP was associated with a nonsignificant decrease in hematoma expansion and thrombotic events. Subgroup analysis of the outcome of efficacy, that is, hematoma expansion signaled a reduced risk with DDAVP. However, DDAVP was associated with a significantly poor neurological outcome in the patients. Until more robust clinical trials are conducted, the use of DDAVP should be considered on a case‐to‐case basis.

## AUTHOR CONTRIBUTIONS

**Faizan Shahzad**: Writing—original draft; writing—review and editing; software; formal analysis. **Usman Ahmed**: Writing—original draft; writing—review and editing; software; formal analysis. **Ayesha Muhammad**: Investigation; methodology; software. **Farhan Shahzad**: Writing—original draft; software; formal analysis; methodology. **Syed Imam Naufil**: Conceptualization; investigation; writing—original draft. **Mohamad Walid Sukkari**: Conceptualization; investigation; writing—review and editing. **Abdullah Bin Kamran**: Conceptualization; investigation; validation; visualization; data curation. **Sara Murtaza**: Conceptualization; investigation; methodology; validation; data curation. **Marwah Khalid**: Conceptualization; investigation; methodology; validation; data curation. **Haroon Shabbir**: Conceptualization; investigation; methodology; validation; data curation. **Sajeel Saeed**: Conceptualization; investigation; methodology; validation; data curation; supervision.

## FUNDING INFORMAION

No funding was received for this research

## CONFLICT OF INTEREST STATEMENT

The authors would like to declare no conflicts of interest.

### PEER REVIEW

The peer review history for this article is available at https://publons.com/publon/10.1002/brb3.3540

## Supporting information

Supplementary Materials

## Data Availability

The data that supports the findings of this study is available in the supplementary material (Supplementary File 1) of this article.
